# Combination therapy with remdesivir and monoclonal antibodies protects nonhuman primates against advanced Sudan virus disease

**DOI:** 10.1172/jci.insight.159090

**Published:** 2022-05-23

**Authors:** Robert W. Cross, Zachary A. Bornholdt, Abhishek N. Prasad, Courtney Woolsey, Viktoriya Borisevich, Krystle N. Agans, Daniel J. Deer, Dafna M. Abelson, Do H. Kim, William S. Shestowsky, Lioudmila A. Campbell, Elaine Bunyan, Joan B. Geisbert, Natalie S. Dobias, Karla A. Fenton, Danielle P. Porter, Larry Zeitlin, Thomas W. Geisbert

**Affiliations:** 1 Galveston National Laboratory and; 2 Department of Microbiology and Immunology, University of Texas Medical Branch, Galveston, Texas, USA.; 3 Mapp Biopharmaceutical, Inc., San Diego, California, USA.; 4 Gilead Sciences, Inc., Foster City, California, USA.

**Keywords:** Virology, Immunotherapy

## Abstract

A major challenge in managing acute viral infections is ameliorating disease when treatment is delayed. Previously, we reported the success of a 2-pronged mAb and antiviral remdesivir therapeutic approach to treat advanced illness in rhesus monkeys infected with Marburg virus (MARV). Here, we explored the benefit of a similar combination therapy for *Sudan ebolavirus* (Sudan virus; SUDV) infection. Importantly, no licensed anti-SUDV therapeutics currently exist, and infection of rhesus macaques with SUDV results in a rapid disease course similar to MARV with a mean time to death of 8.3 days. When initiation of therapy with either remdesivir or a pan-ebolavirus mAb cocktail (MBP431) was delayed until 6 days after inoculation, only 20% of macaques survived. In contrast, when remdesivir and MBP431 treatment were combined beginning 6 days after inoculation, significant protection (80%) was achieved. Our results suggest that combination therapy may be a viable treatment for patients with advanced filovirus disease that warrants further clinical testing in future outbreaks.

## Graphical abstract

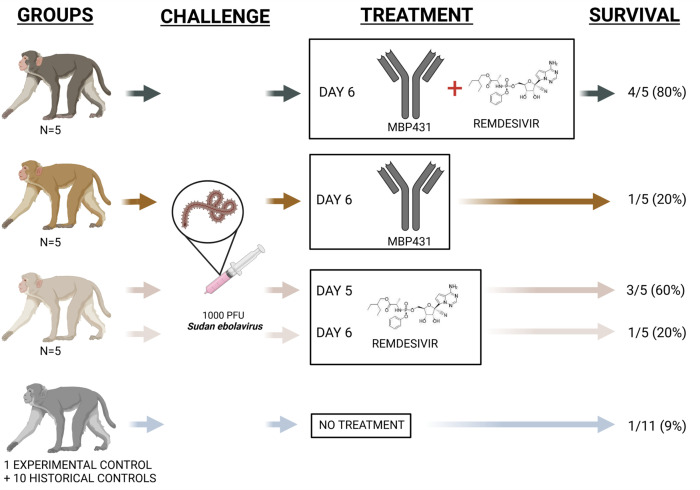

## Introduction

Filoviruses (family *Filoviridae*) are nonsegmented negative sense RNA viruses within the order *Mononegavirales*. Members of 2 genera, *Ebolavirus* and *Marburgvirus*, are known to cause severe and often fatal hemorrhagic fever in humans (Ebola virus disease [EVD] and Marburg virus disease, respectively), with case fatality rates ranging from approximately 23% to 90% (1). Within the genus *Ebolavirus*, 3 species are responsible for nearly all known cases of symptomatic disease: *Zaire ebolavirus* (Ebola virus; EBOV), *Sudan ebolavirus* (Sudan virus; SUDV), and *Bundibugyo ebolavirus* (Bundibugyo virus). Only a single human case of *Tai Forest ebolavirus* (Taï Forest virus) infection has been reported, which presented as EVD and resolved after a period of hospitalization (2, 3). The first known outbreak of SUDV in 1976 began several months before and ran concurrently with the first known outbreak of EBOV; although it occurred in separate (though neighboring) countries, it was initially believed a single etiological agent was responsible for both outbreaks (1, 4). Despite strong circumstantial evidence implicating several species of insectivorous and frugivorous bats, a definitive natural reservoir species has not been identified for any ebolavirus, although the Egyptian rousette bat (*Rousettus aegyptiacus*) is a known reservoir for Marburg virus (MARV) (5). This ambiguity in the identification of a natural reservoir means that outbreaks of SUDV remain impossible to predict. Additionally, as with EBOV and MARV, SUDV has bioweapon potential (6). Therefore, it is of critical importance that in addition to potential preventive vaccines, safe and effective postexposure prophylactics and therapies are available in the event of an SUDV outbreak.

Compared with EBOV and MARV, research into SUDV-specific postexposure therapeutics has been limited. Lipid-encapsulated siRNAs targeting the VP35 mRNA of SUDV completely protected rhesus macaques from lethal infection when treatment was initiated up to 4 days after challenge (7). The small-molecule antiviral favipiravir has shown promising efficacy in small-animal models of SUDV infection when administered as late as 4 days after infection (dpi; refs. 8, 9), although validation in nonhuman primates (NHPs) is lacking. In addition to SUDV-specific mAb cocktails (10), several mAbs or mAb cocktails have shown cross-reactive therapeutic protection against lethal disease from several filoviruses, including SUDV, in small-animal (11) and NHP models (12, 13). Recently, the first pan-ebolavirus mAb cocktail, composed of 2 individual mAbs (MBP134), was demonstrated to confer significant (80%) rescue from lethal SUDV infection in rhesus monkeys when administered as a single dose (i.v.) at 5 days dpi (14). Importantly, in all these studies, therapeutic efficacy was only demonstrated when treatment was initiated by 5 dpi.

Remdesivir (GS-5734) is a monophosphoramidate prodrug of an adenosine nucleoside analog and has shown therapeutic benefit in NHPs against several diverse lineages of RNA viruses, including members of *Filoviridae* (15–17), *Paramyxoviridae* (18), and *Coronaviridae* (19, 20). Remdesivir has also shown inhibitory activity specifically against SUDV in vitro (15). Remdesivir restricts viral replication by impeding synthesis of viral RNA (vRNA) by the vRNA-dependent RNA polymerase via delayed chain termination as well as template-mediated inhibition mechanisms (21, 22). We have previously demonstrated that combining a 12-day course of remdesivir treatment with a single-dose mAb prevented lethal disease in 80% of rhesus macaques challenged with the highly pathogenic Angola variant of MARV (17). Importantly, combining treatments extended the therapeutic window of efficacy from 5 dpi when administered as monotherapies to 6 dpi when administered together.

Here, we investigated the therapeutic efficacy of a once-daily 12-dose remdesivir regimen in a near uniformly lethal rhesus macaque model of SUDV infection. We then evaluated the therapeutic benefit of coadministering remdesivir with the pan-ebolavirus mAb cocktail MBP431 (14) during advanced stages of SUDV disease at a point beyond successful therapeutic intervention for remdesivir alone. In congruence with our earlier report on MARV, our findings here further support the development of protocols that utilize therapeutics with complementary mechanisms of action to extend the window of therapeutic intervention in cases of SUDV infection as well as the general treatment of emerging viruses in human populations.

## Results

### Experimental challenge of rhesus macaques with SUDV and treatment with remdesivir at 5 dpi.

**Figure 1:**
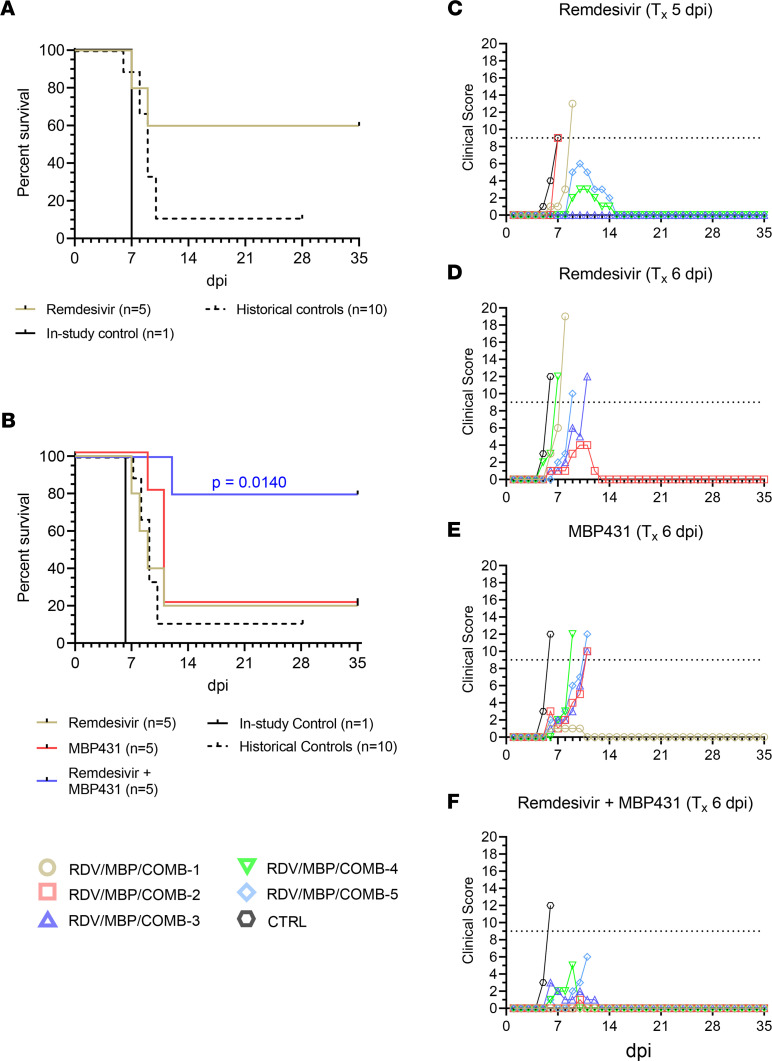
Survival analysis and clinical scoring of rhesus macaques challenged with SUDV. (**A**) Kaplan-Meier survival curves of rhesus macaques receiving treatment beginning 5 dpi (*n* = 5) and untreated in-study (*n* = 1) and historical positive control animals (*n* = 10). (**B**) Kaplan-Meier survival curves of rhesus macaques receiving treatment beginning 6 dpi (*n* = 5 per group) and untreated in-study (*n* = 1) and historical positive control animals (*n* = 10). For **A** and **B**, curves for the in-study control and historical control animals are shown separately; however, for statistical comparisons, the in-study control was pooled with the historical controls. The reported 2-tailed *P* value was derived from the Mantel-Cox log-rank test corrected for multiple comparisons using the Holm-Šídák method and was rounded to 4 decimal places. (**C**–**F**) Clinical scoring for rhesus macaques with treatment initiated 5 dpi with remdesivir (**C**) or with treatment initiated 6 dpi with remdesivir (**D**), MBP431 (**E**), or combined remdesivir/MBP431 (**F**). The horizontal dashed line represents the minimum clinical score by which euthanasia criteria was met. T_x_, treatment.

To establish a temporal threshold for efficacious monotherapeutic treatment of EVD caused by SUDV in rhesus macaques, we challenged a cohort of healthy adult macaques (*n* = 6) with a target dose of 1000 PFU of SUDV (Gulu variant) by i.m. injection. At 5 dpi, the experimental cohort (*n* = 5) received a 10 mg/kg i.v. loading dose of remdesivir followed by 5 mg/kg daily maintenance doses at 6–16 dpi, for a total of 12 consecutive days of treatment, as previously described (16, 17). A single untreated animal served as the in-study positive control. All animals developed fever by 5 dpi, which progressed to severe EVD and clinical scores necessitating humane euthanasia in 2/5 treated animals at 7 and 9 dpi (mean time to death [MTD] = 8.0 ± 1.0 dpi) (Figure 1, A and C, and Supplemental Table 1; supplemental material available online with this article; https://doi.org/10.1172/jci.insight.159090DS1). The in-study control animal was euthanized at 7 dpi. For statistical comparisons, the in-study positive control was grouped with 1 surviving and 9 fatal historical positive control animals challenged using the same virus stock, dose, and challenge route (combined *n* = 11, MTD = 8.3 ± 1.3). The observed survival difference between the remdesivir-treated and control cohorts was not statistically significant (*P* = 0.063, Fisher’s exact test; *P* = 0.084, Mantel-Cox log-rank test). A single surviving animal from the remdesivir-treated group (D5-RDV-3) developed a mild self-limiting febrile illness with a short period of decreased appetite; the remaining 2 animals that survived (D5-RDV-4, D5-RDV-5) developed more severe disease with clinical signs similar to the in-study control and historical controls, including fever, decreased appetite/anorexia, depression, hunched posture, generalized weakness, petechial rash, recumbency, ataxia, edema, and/or diarrhea, before eventually convalescing (Supplemental Table 1). All animals exhibited marked deviation from baseline hematological and serum analyte values compared with baseline (day of challenge), including lymphocytopenia, thrombocytopenia, monocytopenia, neutrophilia, and elevated markers of hepatic/pancreatic injury (e.g., alanine aminotransferase [ALT], aspartate aminotransferase [AST], alkaline phosphatase [ALP], gamma-glutamyltransferase [GGT], serum amylase) and acute systemic inflammation (C-reactive protein [CRP]; Supplemental Table 1 and Supplemental Figure 1). These markers eventually returned to near-baseline values in surviving animals.

### Combining remdesivir with MBP431 enhances protective efficacy and extends the window of treatment for lethal EVD.

Given that remdesivir monotherapy initiated at 5 dpi afforded only partial protection from lethal EVD, we next assessed whether remdesivir in combination with a pan-ebolavirus mAb cocktail, MBP431, which was previously shown to protect when initiated up to 5 dpi (14), could both improve survival outcomes and extend the therapeutic window of efficacy in this model. We conducted a second study with 16 rhesus macaques challenged with SUDV (Gulu variant), but initiation of treatment was delayed to 6 dpi. In addition to remdesivir and MBP431-only treatment groups, the study design included a third cohort of animals treated with a single dose of MBP431 (15 mg/kg i.v.) at 6 dpi in tandem with a 12-day course of remdesivir (6–17 dpi). Monotherapy with either remdesivir or MBP431 resulted in low survival, with only 1/5 (20%) animals from each cohort surviving to the study endpoint (MTD = 8.8 ± 1.5 dpi and 10.5 ± 0.9 dpi, respectively) (Figure 1B). The control animal developed clinical signs of EVD beginning on 5 dpi, including decreased appetite/anorexia, hunched posture, weakness, and petechial rash/ecchymosis, and reached a clinical score requiring humane euthanasia on 6 dpi (Figure 1, D and F, and Supplemental Table 2). Similarly, all subjects in the remdesivir-only, MBP431-only, and combination-treated groups displayed signs of EVD beginning 5 to 6 dpi (Supplemental Table 2). In contrast, 4/5 animals (80%) in the combined remdesivir/MBP431 treatment group survived to the study endpoint (35 dpi). As in the first study, for statistical comparisons of survival, the in-study control from this study was grouped with the historical control animals (*n* = 11, MTD = 8.3 ± 1.3). The difference in survival between the cohort receiving combined remdesivir/MBP431 treatment was statistically significant (Hochberg multiplicity-corrected *P* = 0.038; Fisher’s exact test). Additionally, there was a significant difference in the survival curves for the combination-treated cohort compared with untreated controls (*P* = 0.014, Mantel-Cox log-rank test with Holm-Šídák correction for multiple comparisons) (Figure 1B). In contrast, there was no statistically significant difference in the survival curves for either remdesivir- or MBP431-only treated cohorts compared with untreated controls (*P* = 0.479 and *P* = 0.088, respectively, Mantel-Cox log-rank test with Holm-Šídák correction for multiple comparisons). These data demonstrated that combining remdesivir with a pan-ebolavirus mAb cocktail resulted in enhanced survival benefits well into the acute phase of disease. Moreover, 2/4 surviving animals from this cohort exhibited only mild signs of disease, while both surviving animals from remdesivir- or MBP431-only cohorts developed more severe signs of disease and clinical scoring (Figure 1, D and F, and Supplemental Table 2). Two surviving animals from the combination-treated cohort (D6-COMB-3, D6-COMB-4) and each of the surviving animals from the monotherapy-treated cohorts (D6-RDV-2, D6-MBP-1) developed ocular clouding with keratic precipitates, conjunctivitis, and periorbital edema. This morbidity appeared late in the disease course (~20 dpi), and these surviving subjects exhibited more severe clinical signs during the acute phase of illness than the other 2 surviving animals (D6-COMB-1, D6-COMB-2; Supplemental Table 2). The subject that succumbed in the combined-therapy cohort (D6-COMB-5) developed severe clinical signs of EVD beginning on the day that treatment was initiated and succumbed from the disease on 11 dpi. As in the prior study, all animals exhibited marked deviation from baseline hematological and serum analyte values at 6 dpi typical of SUDV infection in rhesus macaques (Supplemental Figure 2 and Supplemental Table 2). In surviving animals from MBP431-only and combination-treated groups, most markers returned to near-baseline values by 12 dpi, while some markers (e.g., ALT, AST, ALP, GGT) remained slightly elevated beyond this point in the surviving animal from the remdesivir-only cohort.

### Therapeutic reduction of viral load.

**Figure 2:**
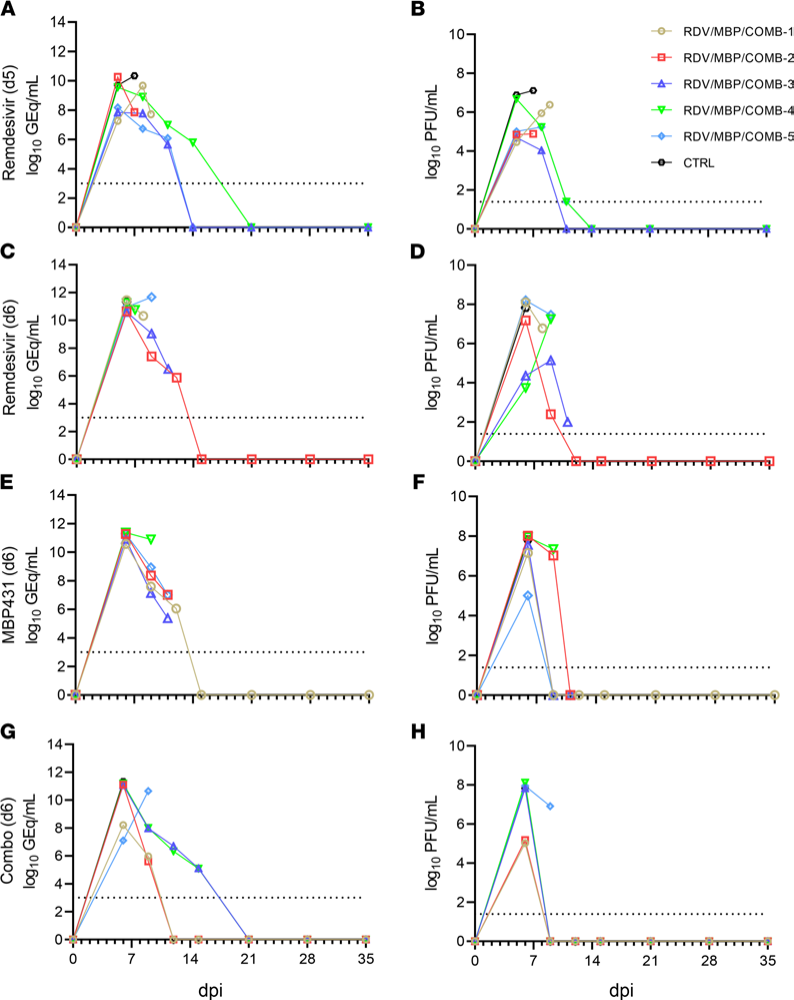
Circulating viral RNA and infectious virus titers from SUDV-challenged rhesus macaques. Viral load was determined by RT-qPCR of whole blood (**A**, **C**, **E**, and **G**) or plaque titration of plasma (**B**, **D**, **F**, and **H**). (**A** and **B**) Treatment at 5 dpi with remdesivir, (**C** and **D**) treatment at 6 dpi with remdesivir, (**E** and **F**) treatment at 6 dpi with MBP431, (**G** and **H**) treatment at 6 dpi with combined remdesivir/MBP431. For all panels, individual data points represent the mean of 2 technical replicates. Dashed horizontal lines indicate the limit of detection (LOD) for the assay (1000 GEq/mL for RT-qPCR; 25 PFU/mL for plaque titration). To fit on a log-scale axis, zero values (below LOD) are plotted as “1” (100).

In the first study, all animals had high levels of circulating SUDV RNA (vRNA) when treatment was initiated at 5 dpi (~10^7^–10^10^ genome equivalents [GEq]), as measured by quantitative reverse transcription PCR (RT-qPCR) (Figure 2A). Likewise, circulating infectious SUDV was recovered by plaque assay from the plasma of all animals beginning 5 dpi (Figure 2B). Animals that did not survive were viremic and had detectable circulating vRNA up to the point they were euthanized. In surviving animals, infectious SUDV titers gradually declined upon initiation of remdesivir treatment, becoming undetectable or nearly so by 11 dpi. Likewise, circulating vRNA gradually declined to undetectable quantities (mean dpi of last positive RT-qPCR result = 12.0 ± 1.4 dpi). vRNA was detected in a panel of selected tissues collected at necropsy. vRNA abundance was higher in animals that succumbed compared with those that survived and was below detection in the pancreata of 2 surviving animals (D5-RDV-3, D5-RDV-5) and the eye from a single animal (D5-RDV-5; Supplemental Figure 3A).

As in the first study, all animals in the second study were viremic when treatment was initiated at 6 dpi (Figure 2, C–H). vRNA abundance in whole blood ranged from approximately 10^7^ to 10^11^ GEq/mL at the time treatment was initiated and was last detected from each of the survivors from the monotherapy cohorts at 12 dpi and from subjects D6-COMB-1 and D6-COMB-2 at 9 dpi (Figure 2, C and E). D6-COMB-4 and D6-COMB-5 had detectable levels of circulating vRNA up to 15 dpi (Figure 2G). Circulating infectious virus titers ranged from approximately 10^4^ to 10^8^ PFU/mL of plasma at the time treatment was initiated (Figure 2, D, F, and H). The severity of viremia at the time treatment was first administered was not predictive of clinical outcome, regardless of treatment. The surviving animal from the MBP431-only treated group and the 4 surviving animals from the combination-treated group exhibited rapid declines in infectious SUDV titers, with the virus becoming undetectable by the following sampling point after treatment was initiated (9 dpi). Three of the animals that developed fatal EVD from the MBP431-only group had undetectable viremia at the terminal time point (11 dpi, Figure 2F). This was hypothesized to be due to circulating MBP431 interfering with the plaque assay through neutralization of the virus, which suggests that viral damage leading to a lethal outcome may have been in tissues with less mAb and/or more virus than in circulation. vRNA was detected in most or all tissues from surviving animals in the monotherapy groups (Supplemental Figure 3, B and C), but was absent in several tissues, including potential reservoir sites (e.g., eyes, gonads) from some combination-treated animals (Supplemental Figure 3D). Detectable vRNA was notably absent from the liver of 3 of the 4 surviving combination-treated animals (D6-COMB-1, D6-COMB-2, D6-COMB-3) and the surviving animal from the MBP431-treated group (D6-MBP-1).

### Gross lesions and histopathology.

**Figure 3:**
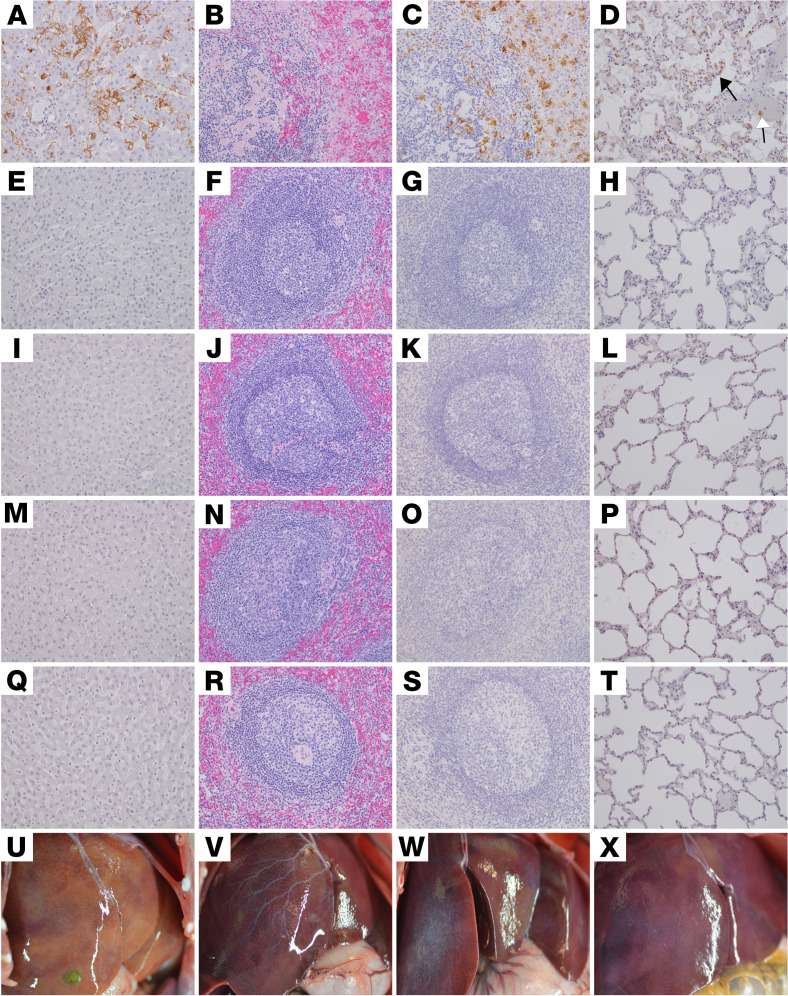
Pathology of the liver, spleen, and lung of SUDV-challenged rhesus macaques. Representative photomicrographs of IHC for anti-SUDV VP 40 antigen (brown) in liver (**A**, **E**, **I**, **M**, and **Q**), spleen (**C**, **G**, **K**, **O**, and **S**), and lung (**D**, **H**, **L**, **P**, and **T**). All photomicrographs were taken at 20× original magnification. H&E staining of the spleen (**B**, **F**, **J**, **N**, and **R**) and gross images of the liver (**U**, **V**, **W**, and **X**. D5-CTRL (**A**–**D**). IHC positivity of sinusoidal lining cells, Kupffer cells, and rarely, hepatocytes (**A**); marked disruption of normal splenic architecture with fibrin accumulation, hemorrhage, and lymphocytolysis of the white pulp (**B**); IHC-positive mononuclear cells within the red and white pulp of the spleen (**C**); IHC-positive mononuclear cells within the alveolar septa (black arrow) and alveolar macrophages (white arrow) (**D**). No appreciable immunolabeling or lesions noted in the liver, spleen, or lung of D5-RDV-4 (**E**–**H**), D6-RDV-2 (**I**–**L**), D6-MBP-1 (**M**–**P**), and D6-COMB-3 (**Q**–**T**). Necrotizing hepatitis described as multifocal to coalescing hepatic pallor in D6-COMB-5 that succumbed from SUDV infection (**U**). No appreciable gross lesions noted in the liver of representative animals at the study endpoint for D6-RDV-2 (**V**), D6-MBP-1 (**W**), and D6-COMB-3 (**X**).

Necropsy was performed on all macaques after euthanasia. Lesions consistent with SUDV infection were present in animals that succumbed from the disease despite treatment (D5-CTRL, D5-RDV-1, D5-RDV-2, D6-CTRL, D6-RDV-1, D6-RDV-2, D6-RDV-3, D6-RDV-4, D6-RDV-5, D6-MBP-2, D6-MBP-3, D6-MBP-4, D6-MBP-5, and D6-COMB-5). Gross lesions present in all that succumbed from SUDV infection included necrotizing hepatitis (Figure 3U), pneumonia, splenomegaly, and lymphadenomegaly. Additional gross lesions noted in some subjects included adrenomegaly (D5-CTRL, D5-RDV-1, D5-RDV-2, D6-CTRL, D6-RDV-1, D6-RDV-2, D6-RDV-3, D6-RDV-4, D6-RDV-5, D6-MBP-2, D6-MBP-4, D6-MBP-5, and D6-COMB-5), petechial skin rash or edema (D5-CTRL, D5-RDV-2, D6-CTRL, D6-RDV-1, D6-RDV-2, D6-RDV-3, D6-RDV-4, D6-RDV-5, D6-MBP-2, D6-MBP-4, D6-MBP-5, and D6-COMB-5), intestinal hemorrhage (D6-CTRL, D6-RDV-1, D6-RDV-4, D6-RDV-5, D6-MBP-2, D6-MBP-4, and D6-COMB-5), testicular hemorrhage (D5-RDV-1 and D6-RDV-3), ascites (D5-RDV-1, D6-CTRL, and D6-COMB-5), and pleural effusion (D6-CTRL). Additional unique lesions of anterior uveitis and cataracts were noted in 3 surviving macaques that were examined at necropsy (D6-RDV-2, D6-MBP-1, and D6-COMB-3). No gross lesions were apparent in the 9 subjects that were examined at the study endpoint (D5-RDV-3, D5-RDV-4, D5-RDV-5, D6-RDV-2, D6-MBP-1, D6-COMB-1, D6-COMB-2, D6-COMB-3, and D6-COMB-4, Figure 3, V, W, and X).

**Figure 4:**
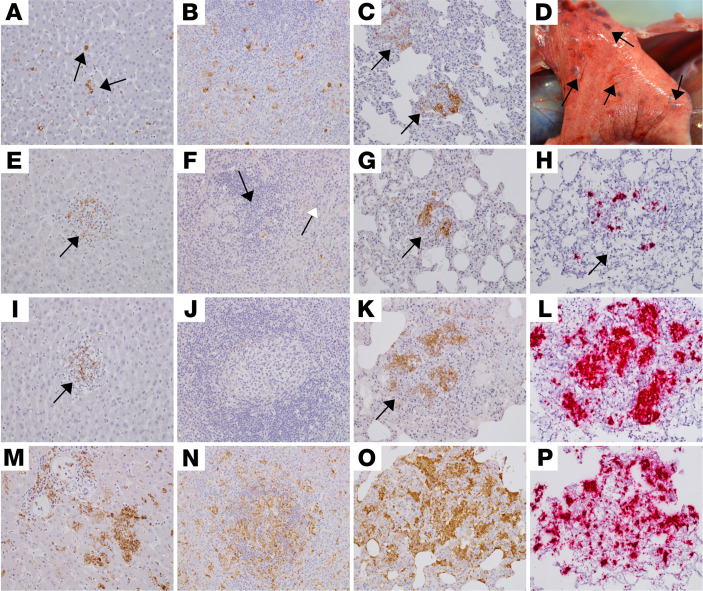
Pathology of the liver, spleen, and lung of SUDV-challenged rhesus macaques that succumbed at 9–12 dpi. Representative photomicrographs of IHC at 20× original magnification for SUDV VP40 antigen (brown) in the liver (**A**, **E**, **I**, and **M**), spleen (**B**, **F**, **J**, and **N**), and lung (**C**, **G**, **K**, **O**). IHC positivity for rare Kupffer cells/mononuclear cells (black arrows) in the liver of D5-RDV-1 (**A**), small clusters in D6-RDV-3 (**E**) and D6-MBP-3 (**I**), and moderate clusters in D6-COMB-5 (**M**). IHC positivity for scattered mononuclear cells within the red and white pulp of the spleen for D5-RDV-1 (**B**), rarely the white pulp (black arrow) and red pulp (white arrow) D6-RDV-3 (**F**), and moderately in D6-COMB-5 (**N**). No appreciable immunolabeling was present in the spleen of D6-MBP-3 (**J**). Mononuclear cells had IHC positivity within clustered/nodular lesions of the lung of D5-RDV-1 (**C**, arrows), D6-RDV-3 (**G**, arrow), D6-MBP-3 (**K**, arrow), and D6-COMB-5 (**O**). Multifocal raised white nodules consistent with pneumonia on histology were present in D6-COMB-5 at gross examination (**D**). Representative positive ISH (red) at 20× original magnification of pulmonary nodules for D6-RDV-3 (**H**), D6-MBP-3 (**L**), and D6-COMB-5 (**P**).

Five of 11 SUDV-infected animals (D6-RDV-1, D6-RDV-4, D6-RDV-5, D6-MBP-4, and D6-COMB-5) that succumbed from the disease across all treatment groups displayed lesions consistent with SUDV infection and were similar in severity to the 2 positive controls (D5-CTRL and D6-CTRL). Significant lesions in these macaques included necrotizing hepatitis with fibrin deposition in sinusoids, necrotizing splenitis with fibrin deposition (Figure 3B), and interstitial pneumonia with pulmonary edema. Less pronounced inflammatory lesions were also present in multiple organs. These included lymphoid medullary histiocytosis (axillary and inguinal), pancreatitis, conjunctivitis, nephritis, adrenalitis, uveitis, cystitis, metritis, prostatitis, and oophoritis or epididymitis. All lesions had colocalized immunolabeling for anti-SUDV VP40 antigen within mononuclear inflammatory cells, endothelium, and rarely, epithelial cells (Figure 3, A, C, and D, and Figure 4, M, N, and O).

Six of 11 SUDV-infected animals (D5-RDV-1, D5-RDV-2, D6-MBP-5, D6-RDV-3, D6-MBP-2, and D6-MBP-3) that succumbed from the disease across all treatment groups displayed mild lesions consistent with SUDV infection. Anti-SUDV VP40 IHC positivity was observed in clusters of mononuclear cells within the liver (Figure 4, A, E, and I) and lung (Figure 4, C, G, and K), and in rare mononuclear cells within the red and white pulp of the spleen (Figure 4, B and F). SUDV vRNA was detected by ISH in clustered mononuclear cells from the lungs of subject D6-RDV-3 (Figure 4H). Immunolabeling was not detected in the spleen from subject D6-MBP-3 (Figure 4J). Nodular pneumonia composed of necrotizing pyogranulomas inflammation was unique to 4 subjects (D6-MBP-5, D6-MBP-2, D6-MBP-3, and D6-COMB-5; Figure 4, D, K, and O). Mononuclear cells within the pyogranulomas were positive by IHC (Figure 4, K and O) and in situ hybridization (ISH; Figure 4, L and P) in 4 animals (D6-MPB-2, D6-MBP-3, D6-MBP-5, and D6-COMB-5).

At the study endpoint (35 dpi), no histologic lesions or immunolabeling was present in the examined tissues consistent with SUDV disease in 6/9 (67%) surviving macaques: D5-RDV-5, D5-RDV-3, D5-RDV-4 (Figure 3, E–H), D6-COMB-1, D6-COMB-2, and D6-COMB-4. One surviving animal from each 6 dpi treatment group had inflammatory ocular lesions with chromogenic labeling of vRNA but lacked histologic lesions and immunolabeling in all other organs: D6-RDV-2 (Figure 3, I–L), D6-MBP-1 (Figure 3, M–P), and D6-COMB-3 (Figure 3, Q–T). No ocular lesions were noted in 6 of 9 of the surviving macaques: D5-RDV-5, D5-RDV-3, D5-RDV-4, D6-COMB-1, D6-COMB-2, and D6-COMB-4.

## Discussion

The ongoing SARS-CoV-2 pandemic has emphasized the necessity for the development of vaccines and postexposure treatments for emerging and reemerging viral agents. Although SUDV has caused fewer outbreaks and resulted in far fewer clinical cases and deaths than EBOV, it likely shares a similar ecological niche and may be more geographically widespread than is currently appreciated. The lack of knowledge regarding its animal reservoir, and thus the unknown distribution of the virus, foreshadows the possibility of large-scale outbreaks unexpectedly arising in underprepared regions, akin to the 2013 to 2016 EBOV epidemic in West Africa. Moreover, the 2018–2020 EBOV outbreak in the Democratic Republic of Congo (DRC) demonstrated that even in countries with significant experience in dealing with filovirus outbreaks, containment measures may rapidly deteriorate due to a variety of sociopolitical, infrastructural, and cultural factors, potentially leading to thousands of cases and fatalities. Therefore, in the absence of population-wide vaccination, it is imperative that a toolbox of rapidly deployable, highly efficacious therapeutics for SUDV infection be developed.

Both mAbs and broad-spectrum small-molecule antivirals have been the focus of much of the recent research and development into therapies for emerging viral infections. In response to the ongoing SARS-CoV-2 pandemic, several mAbs or cocktails of mAbs have been developed, assessed, and authorized for use under compassionate/emergency use criteria (23–26), including as a preexposure prophylactic (27). Additionally, a variety of existing small-molecule therapeutics not previously purposed as antivirals have been assessed for their potential to treat COVID-19, with several highly publicized candidates, such as chloroquine/hydroxychloroquine and ivermectin, failing to show significant clinical benefit in large-scale randomized clinical trials and meta-analyses (28–32). In October 2020, after demonstrating positive results in treating patients hospitalized with COVID-19 in phase III clinical trials (33), remdesivir became the first therapeutic to receive FDA approval for the treatment of COVID-19 (34). More recently, molnupiravir (Merck) and nirmatrelvir/ritonavir (Pfizer) have been shown to have a positive clinical benefit in unvaccinated, nonhospitalized adults with COVID-19 when administered orally (35, 36) and have received emergency use authorization from the FDA (37, 38).

Given the rarity of filovirus infections in humans compared with SARS-CoV-2, considerably less attention has been given to the development of vaccines and therapeutics to combat infections by these agents. Early studies into the use of Ig to mitigate EVD showed initial promise in vitro (39, 40) and in small-animal models (41) but failed to provide protection from lethal disease in NHPs (42–45). A subsequent study demonstrated that passive immunotherapy through multiple administrations of purified polyclonal IgG from EBOV- or MARV-vaccinated NHPs surviving challenge could provide complete protection from lethal disease to filovirus-naive NHPs when administered up to 48 hours after challenge (46). The sheer magnitude and duration of the 2013 to 2016 EBOV epidemic in West Africa renewed widespread interest from global health authorities in supporting research and development of effective therapeutics to address future outbreaks (47). During the end of the epidemic, a clinical trial showed that a mAb cocktail, ZMapp, was 92% more effective in reducing mortality compared with supportive care (48). Subsequently, during the 2018 to 2020 DRC EBOV outbreak, the PALM trial concluded that 2 other mAb products, mAb114 and REGN-E3, were more effective than ZMapp and remdesivir at reducing mortality from EVD (49).

We recently reported that combining remdesivir with the MARV-specific mAb MR186-YTE conferred a significant survival benefit to rhesus macaques infected with the Angola isolate of MARV versus monotherapy with either treatment alone (17). Importantly, in addition to a reduction in clinical signs and pathology, combination therapy extended the effective window of treatment during the crucial acute phase of disease (~5–9 dpi), during which, barring intervention, the clinical outcome is likely to be determined. Encouraged by the results of this study, we asked whether an identical approach could be used to treat NHPs infected with another filovirus of concern, SUDV. Treatment with remdesivir alone provided only partial protection (60%) to NHPs when administered at 5 dpi, and only 20% survival was observed when either remdesivir or the pan-ebolavirus mAb cocktail MBP431 were administered as monotherapies at 6 dpi. In contrast, a combined remdesivir/MBP431 treatment protocol dramatically improved survival (80%) when initiated at 6 dpi, and nearly completely ameliorated signs of disease in 50% of surviving animals. Subjects D6-COMB-3 and D6-COMB-4 developed ocular morbidities after convalescence, manifesting as periorbital edema, keratic precipitates, uveitis, and conjunctivitis, which persisted to or close to the study end. Interestingly, these same morbidities were observed in each of the surviving subjects from the monotherapy cohorts (D6-RDV-2 and D6-MBP-1). This implies that above a certain viral burden, even combined therapy may still result in incomplete clearance of the virus and result in the virus remaining in “sanctuary” sites lacking typical immune surveillance. This is supported by the delayed clearance of detectable circulating vRNA in these animals (12–15 dpi). Indeed, uveitis and retinitis have been described in NHPs persistently infected with EBOV after surviving the acute phase of disease (50). Additionally, other studies have described viral persistence in immune-privileged sites (e.g., eyes, testes, brain) in NHPs surviving filovirus challenge (51, 52), with CD68^+^ circulating and tissue-resident (e.g., synovial) macrophages being suggested as a possible reservoir for persistent infection (50, 51, 53). A recent report described a case of fatal apparent recrudescence in a rhesus macaque after treatment with an anti-EBOV mAb cocktail (52). In this study, the macaque exhibited only mild clinical signs of EVD after treatment, and then developed severe EVD and succumbed from the disease approximately 2 weeks later. Given that remdesivir and MBP431 exhibit mechanistically distinct modes of action to inhibit virus replication, we posit that the combined use of both therapeutics likely contributes to the observed survival benefit in SUDV-infected NHPs. In addition, the bioavailability of each therapeutic to different tissues and sites of infection may differ, owing to their disparate physicochemical properties, and thus might mitigate against virus spread more broadly across different body compartments and tissues. Future studies should seek to more comprehensively assess incomplete clearance/persistence in NHP models of filovirus after exposure therapy.

Virus persistence at immune-privileged sites is thought to contribute to various short-lived and long-term sequelae in human survivors of EVD. Prevalent sequelae in survivors include musculoskeletal (38%), neurosensory (37%), and ophthalmic (18%) complications, collectively referred to as “post-Ebola virus disease syndrome” (54). For example, similar to animals in this study, uveitis has also been reported in a human EVD survivor (55). In addition to intensive supportive care, this patient was treated with a siRNA antiviral agent (TKM-100802; Tekmira Pharmaceuticals) and convalescent plasma. Fourteen weeks after disease onset and 9 weeks after the clearance of viremia, viable EBOV was isolated from aqueous humor obtained from the inflamed eye, an immune-privileged site. Whether continuous administration of antivirals or mAb cocktails are effective in preventing ocular disorders or clearing the virus from the eye after recovery is currently unknown. Findings from our study support a postexposure combinational therapeutic study design, which may facilitate evaluation of prolonged therapies targeting complete viral clearance into convalescence.

A primary concern regarding the use of therapeutic mAbs to treat viral infections is the possibility of generating antibody escape mutants. Because most viruses that cause acute infections in humans, such as EBOV and MARV, are likely to be transmitted to naive individuals prior to the infective host mounting a humoral response, the mutational tolerance and capacity to rapidly escape therapeutic intervention with individual mAbs may be underestimated (56). To counter this, mAb therapies are often formulated as cocktails of 2 or more individual mAbs, with the rationale that while the generation of mutants that can escape neutralization by a single mAb may be likely, selection for mutants that can escape cocktails targeting multiple epitopes is far less so (57, 58). Therefore, combining remdesivir with a mAb cocktail likely imposes additional replicative pressures on the virus, which may lessen the likelihood of antibody escape.

Here, we expand upon our findings in the MARV model of infection in rhesus monkeys (17) by demonstrating efficacious treatment of advanced EVD caused by SUDV in rhesus macaques. Collectively, the results of these studies suggest that combining therapeutics with complementary, nonoverlapping modes of action provides better clinical outcomes compared with monotherapy and may help mitigate future outbreaks caused by filoviruses and other emerging viruses.

## Methods

### Virus.

SUDV isolate 200011676 (Gulu variant) originated from a 35-year-old male patient who had died during the 2000–2001 SUDV outbreak in Uganda. The study challenge material was from the second Vero E6 cell passage of SUDV isolate 200011676. Briefly, the first passage at University of Texas Medical Branch (UTMB) consisted of inoculating CDC 808892 (CDC passage 1 of SUDV isolate 200011676) at an MOI of 0.001 onto Vero E6 cells. The cell supernatants were subsequently harvested at 7 dpi and stored at –80°C as approximately 1 mL aliquots. No detectable mycoplasma or endotoxin levels were measured (<0.5 EU/mL). This SUDV stock matched the parent isolate 808892 consensus sequence (RefSeq NC_006432); the 7U percentage was approximately 92.33%.

### NHP challenge and treatment.

Details of the study design for each experiment are provided in Results. All procedures involving physical manipulation were performed under sedation with ketamine. For the first study, 6 healthy adult rhesus macaques (*Macaca mulatta*) (PreLabs) of Chinese origin ranging in age from approximately 4 to 8 years and weighing approximately 4.0 to 6.9 kg were challenged (i.m. in the left quadricep) with a 1000 PFU target dose (actual dose 800 PFU) of SUDV (Gulu variant). Assignment to the treatment group or control was determined prior to challenge by randomization, with effort made to maintain a balanced sex ratio. Treatment with remdesivir was initiated at 5 dpi as a multidose protocol. The duration of this study was 35 days. The second study involved 16 healthy adult rhesus macaques ranging in age from approximately 3 to 7 years and weighing approximately 3.3 to 8.5 kg. Virus challenge was performed identically to the first study with the exception of the actual dose of the virus inoculum (894 PFU). Assignment to each treatment group or control was determined prior to challenge by randomization, with effort made to maintain a balanced sex ratio. Treatment was initiated at 6 dpi as either a single dose (MBP431) or multidose (remdesivir) protocol. The duration of this study was 35 days. The macaques were monitored daily and scored for disease progression with an internal SUDV humane endpoint scoring sheet approved by the UTMB IACUC. The scoring changes measured from baseline included posture and activity level; attitude and behavior; food intake; respiration; and disease manifestations, such as visible rash, hemorrhage, ecchymosis, or flushed skin. A score of 9 or higher indicated that an animal met the criteria for euthanasia.

### Drug formulation.

CHOK1-AF cells stably expressing the ADI-23774^YTE^ and ADI-15878^YTE^ mAbs were generated as previously described (14). Briefly, a dual plasmid system containing expression cassettes for the heavy and light chains of the target mAbs were cotransfected by random integration via chemical means into a modified CHOK1 host cell line (CHOK1-AF), which yielded afucosylated glycans on expressed mAbs. Stable selection was initiated with the inclusion of methionine sulfoximine as a selection agent 24 hours after transfection. Upon completion of the final expansion, the culture was maintained in fed-batch for 14 days, after which the supernatant was clarified via filtration and subsequently sterile-filtered (0.2 μm) into a 20 L bioprocess bag (Thermo Fisher Scientific) prior to protein A purification. MBP431 was then formulated at 22.3 mg/mL in 10 mM histidine, 5% sorbitol, 0.02% PS80, pH 6.0 for i.v. dosing in the challenge study.

Remdesivir was synthesized at Gilead Sciences, Inc. The chemical identity and sample purity were established using NMR, high-resolution mass spectrometry, and HPLC analyses (15). Small-molecule x-ray crystallographic coordinates and structure factor files have been deposited in the Cambridge Structural Database (http://www.ccdc.cam.ac.uk/); accession numbers have been supplied previously (15). Remdesivir drug substance batch number 5734-BC-1P was solubilized in 12% sulfobutylether-β-cyclodextrin in water at pH 3.5, and matching vehicle solution was provided to UTMB for these studies.

### Hematology and serum biochemistry.

Total WBC counts, WBC differentials, RBC counts, platelet counts, hematocrit values, total hemoglobin concentrations, mean cell volumes, mean corpuscular volumes, and mean corpuscular hemoglobin concentrations were analyzed from blood collected in tubes containing EDTA using a Vetscan HM5 laser-based hematologic analyzer (Zoetis). Serum samples were tested for concentrations of albumin, amylase, ALT, AST, ALP, blood urea nitrogen (BUN), calcium, creatinine, CRP, GGT, glucose, total protein, and uric acid by using a Piccolo point-of-care analyzer and Biochemistry Panel Plus analyzer discs (Abaxis).

### RNA isolation from SUDV-infected macaques.

On procedure days, 100 μL of blood from K2-EDTA collection tubes was collected prior to centrifugation and was added to 600 μL of AVL viral lysis buffer with 6 μL carrier RNA (Qiagen, 52906) for RNA extraction. For tissues, approximately 100 mg was stored in 1 mL RNAprotect (Qiagen, 1018087) for at least 4 days for stabilization. RNAprotect was completely removed, and tissues were homogenized in 600 μL RLT buffer (Qiagen, 74004) and 1% β-mercaptoethanol in a 2 mL cryovial using a TissueLyser II (Qiagen, 85300) and 0.2 mm ceramic beads. The tissues sampled included axillary and inguinal lymph nodes, liver, spleen, kidney, adrenal gland, lung, pancreas, urinary bladder, ovary or testis, and eye. All blood samples were inactivated in AVL viral lysis buffer, and tissue samples were homogenized and inactivated in RLT buffer prior to removal from the biosafety level 4 (BSL-4) laboratory. Subsequently, RNA was isolated from blood using the QIAamp viral RNA kit (Qiagen, 52906) and from tissues using the RNeasy Mini kit (Qiagen, 74004) according to the manufacturer’s instructions supplied with each kit.

### Quantification of viral load.

SUDV RNA was detected using the CFX96 detection system (Bio-Rad Laboratories) in 1-step probe RT-qPCR kits (Qiagen, 210212) with the following cycle conditions: 50°C for 10 minutes, 95°C for 10 seconds, 40 cycles of 95°C for 10 seconds, and 59°C for 30 seconds and the following primer/probe sequences: forward: 5′-TCAAATATTGCAACCAATGCTATG-3′; reverse: 5′-GCATGTAACATTGCGGAATTAGG-3′; probe: 6-carboxyfluorescein (6FAM)-5′-CATCCAATCAAAGACATTGCG A′-6 carboxytetramethylrhodamine (TAMRA) (Life Technologies). Threshold cycle values representing SUDV *L* genomes were analyzed with CFX Maestro Software, and data are shown as GEq. To create the GEq standard, RNA from SUDV stocks was extracted, and the number of SUDV *L* genomes was calculated using Avogadro’s number and the molecular weight of the SUDV genome. Limit of detection was 1 × 10^3^ GEq/mL.

Virus titration was performed by plaque assay using Vero E6 cells (ATCC, CRL-1586) from all plasma and tissue samples as previously described (59). Briefly, increasing 10-fold dilutions of the samples were adsorbed to Vero E6 cell monolayers in duplicate wells (200 μL) and overlaid with 0.8% agarose in 1× MEM with 5% FBS and 1% penicillin/streptomycin. After 6 days of incubation at 37°C/5% CO_2_, neutral red stain was added and plaques were counted after 48 hours of incubation. The limit of detection for this assay was 25 PFU/mL.

### Histopathology, IHC, and ISH.

Necropsy was performed. Tissue samples of all major organs were collected for histopathological and IHC examination, immersion-fixed in 10% neutral buffered formalin, and processed for histopathology as previously described (7). Briefly, tissue sections were deparaffinized and rehydrated through xylene and graded ethanols. Slides went through heat antigen retrieval in a steamer at 95°C for 20 minutes in citrate buffer, pH 6.0, 10× (MilliporeSigma). To block endogenous peroxidase activity, slides were treated with 3% hydrogen peroxide and rinsed in distilled water. The tissue sections were processed for IHC using the Thermo Fisher Scientific Autostainer 360. Sequential 15-minute incubations with avidin D and biotin solutions (Vector Laboratories, SP-2001) were performed to block endogenous biotin reactivity. Specific anti-SUDV VP40 immunoreactivity was detected using an anti-SUDV VP40 rabbit primary polyclonal antibody (IBT Bioservices, 0302-001) at a 1:4000 dilution for 60 minutes. Secondary antibody used was biotinylated goat anti-rabbit IgG (Vector Laboratories, BA-1000) at 1:200 for 30 minutes followed by HRP streptavidin, ready to use (Vector Laboratories, SA-5704) for 30 minutes. Slides were developed with DAB chromogen (Dako, K3468) for 5 minutes and counterstained with hematoxylin for 45 seconds.

SUDV RNA (ISH) for FFPE tissues was performed using the RNAscope 2.5 high-definition RED kit (Advanced Cell Diagnostics) according to the manufacturer’s instructions. Advanced Cell Diagnostics designed and produced the 30 ZZ probe pairs targeting the genomic SUDV nucleoprotein (*NP*) gene (catalog 479281). After sectioning, deparaffinization with xylene and graded ethanol washes was performed, followed by peroxidase blocking with hydrogen peroxide. The sections were then heated in RNAscope Target Retrieval reagent buffer (Advanced Cell Diagnostics, 322000) for 35 minutes and then air-dried overnight. The sections were digested with Protease IV (Advanced Cell Diagnostics, 322336) at 40°C in the HybEZ oven (Advanced Cell Diagnostics, 321711) for 25 minutes followed by ISH target probe and incubated at 40°C in the HybEZ oven for 2 hours. After rinsing, the signal was amplified using the company-provided preamplifier and amplifier conjugated to ALP and incubated with a red substrate-chromogen solution for 10 minutes. The sections were then counterstained with hematoxylin for 2 minutes, air-dried, and cover-slipped.

Relative severity scores for histological lesions/immunoreactivity were assigned by a board-certified veterinary pathologist. Representative photomicrographs were qualitatively considered to display lesions that were nominally or ordinally measured by masking of the veterinary pathologist after the examination and ranking lesions to satiate the study objectives, as previously established (60). Additionally, a thorough examination of the target tissues was performed multiple times in a timely manner to maintain interpretation consistency.

### Data availability.

The data sets used and/or analyzed during the current study are available from the corresponding author, TWG, on reasonable request.

### Statistics.

For statistical comparisons, survival data from 10 historical positive control rhesus macaques from both published (7, 14) and unpublished studies, and challenged via the same route with the same virus stock and dose, were added to the in-study control cohorts. All statistical analyses were performed in GraphPad Prism v9.3.1, except for the Hochberg step-up correction for multiple comparisons, which was performed in R (61) using the “p.adjust” function of the included stats v.4.0.2 package. Unless otherwise indicated, all reported *P* values (α = 0.05) are 2 tailed and rounded to 4 decimal places.

### Study approval.

Animal studies were performed in BSL-4 biocontainment at the UTMB and approved by the UTMB Institutional Biosafety Committee and IACUC. Animal research was conducted in compliance with UTMB IACUC, Animal Welfare Act, and other federal statutes and regulations relating to animals. The UTMB animal research facility is fully accredited by the Association for Assessment and Accreditation of Laboratory Animal Care and adheres to principles specified in the eighth edition of the *Guide for the Care and Use of Laboratory Animals* (National Academies Press, 2011).

## Author contributions

RWC, ZAB, LZ, DPP, and TWG conceived and designed the study. DMA, DHK, WSS, and LAC manufactured the MBP431. EB prepared remdesivir formulations. DJD, JBG, and TWG performed the NHP infection and treatment experiments. RWC, ANP, CW, DJD, JBG, and TWG conducted clinical observations of the animals. VB and KNA performed the clinical pathology assays. VB performed the SUDV infectivity assays. KNA performed the PCR assays. NSD performed the IHC and ISH assays. KAF performed histological and IHC analysis of the data. All authors analyzed the data. ANP wrote the paper. RWC, ZAB, CW, KAF, LZ, DPP, and TWG edited the paper. All authors had access to the data and approved the final version of the manuscript.

## Supplementary Material

Supplemental data

## Version 1 04/12/2022

In-Press Preview

## Version 2 05/23/2022

Electronic publication
