# Supplementary material for: Combination therapy with remdesivir and monoclonal antibodies protects nonhuman primates against advanced Sudan virus disease
Source: JCI Insight. 2022 May 23;7(10):e159090. doi: 10.1172/jci.insight.159090 (PMC9220838; doi:10.1172/jci.insight.159090)
Supplement: Supplemental data [file jciinsight-7-159090-s093.pdf]

Supplementary Table 1. Clinical description and outcome of rhesus macaques following SUDV challenge with remdesivir treatment initiated 5 dpi.

NHP	Sex	Treatment	Clinical illness	Clinical pathology
D5-RDV-1	M	Remdesivir	Fever (d5); decreased appetite (d6, 7); anorexia (d8, 9); petechial rash (d6-9); hunched posture (d9); depression (d9); weakness (d9); ataxia (d9); recumbency (d9); diarrhea (d9); emesis (d9); tachypnea (d9). Subject euthanized (d9).	Lymphocytopenia (d5, 8, 9); thrombocytopenia (d5, 8, 9); monocytopenia (d8); basopenia (d8, 9); monocytosis (d5); neutrophilia (d5, 8, 9); eosinophilia (d5, 9); basophilia (d5); hypoglycemia (d5, 8); hyperglycemia (d9); hypoalbuminemia (d8, 9); hypoamylasemia (d5, 8, 9); ALT ↑ (d8); AST ↑ (d5, 9), ↑↑(d8); ALP ↑ (d8, 9); GGT ↑ (d8); CRP ↑↑↑↑ (d5), ↑ (d8), ↑↑↑ (d9).
D5-RDV-2	F	Remdesivir	Fever (d5-7); decreased appetite (d1, 5-7); depression (d7); weakness (d7); recumbency (d7). Subject euthanized (d7).	Lymphocytopenia (d5, 7); monocytopenia (d5); monocytosis (d7); neutrophilia (d5, 7); eosinophilia (d7); basophilia (d5, 7); hypoglycemia (d7); hypoamylasemia (d5, 7); CRE ↑ (d7); ALT ↑ (d7); AST ↑ (d7); ALP ↑ (d5, 7); CRP ↑↑↑ (d5, 7).
D5-RDV-3	M	Remdesivir	Fever (d5-7); decreased appetite (d7, 9, 10). Subject survived to study endpoint (d35).	Lymphocytopenia (d5); thrombocytopenia (d8, 11, 14, 35); monocytopenia (d5, 11, 14, 21, 35); lymphocytosis (d11); neutrophilia (d5, 8, 11, 14, 21, 35); eosinophilia (d5, 11, 14, 21, 35); basophilia (d5, 14); hypoalbuminemia (d8, 11, 14); hypoamylasemia (d5); AST ↑ (d8, 11); CRP ↑↑↑↑ (d5), ↑ (d8).
D5-RDV-4	F	Remdesivir	Fever (d5, 9); decreased appetite (d6-8, 10, 12-14, 24); anorexia (d9, 11); petechial rash (d7); hunched posture (d11-14); ataxia (d11-18); facial edema (d13-14). Subject survived to study endpoint (d35).	Lymphocytopenia (d5, 8, 11); thrombocytopenia (d8, 11); monocytopenia (d8, 11, 14); monocytosis (d5); neutrophilia (d5, 8, 11, 14, 35); eosinophilia (d11); basophilia (d11); hypoalbuminemia (d11, 14, 21); hypoamylasemia (d5, 8, 11); CRE ↑ (d8, 11); AST ↑ (d8, 11); CRP ↑↑↑↑ (d5), ↑ (d8, 11).
D5-RDV-5	M	Remdesivir	Fever (5, 9); decreased appetite (d5-8, 15-17); anorexia (d9-14); weakness (d9, 11-17); recumbency (d9); hunched posture (d10-14); depression (d11-14); diarrhea (d14-16); ataxia (d11-17); loose stool (d21). Subject survived to study endpoint (d35).	Lymphocytopenia (d5, 8, 11, 14, 21); thrombocytopenia (d11); monocytopenia (d5, 8, 11, 14, 21); neutrophilia (d5, 8, 11, 14, 21, 35); eosinophilia (d5, 11, 14, 21); basopenia (d5, 11, 14, 21); hypoalbuminemia (d8, 11, 14, 21); hypoamylasemia (d5, 8, 11, 14); BUN ↑ (d11); CRE ↑ (d8, 11); AST ↑ (d8, 11, 14); ALP ↑ (d14, 21); CRP ↑↑↑↑ (d5, 11), ↑ (d8), ↑↑ (d14).
D5-CTRL	F	In-Study Control	Fever (d5); decreased appetite (d1, 2, 4); anorexia (d5-7); petechial rash (d6, 7); depression (d7); weakness (d7); recumbency (d7); unresponsiveness (d7). Subject euthanized (d7).	Lymphocytopenia (d5); thrombocytopenia (d7); monocytosis (d5, 7); neutrophilia (d5, 7); eosinophilia (d5); basophilia (d5); hypoalbuminemia (d7); hypoamylasemia (d5, 7); CRE ↑ (d7); ALT ↑ (d7); AST ↑ (d5), ↑↑↑ (d7); ALP ↑ (d5), ↑↑↑ (d7); GGT ↑ (d7); CRP ↑↑↑↑ (d5, 7)

Days after SUDV challenge are in parentheses. All reported findings are in comparison to baseline (d0) values. Decreased appetite is defined as some food but not all food consumed from the previous day. Anorexia is defined as no food consumed from the previous day. Fever is defined as a temperature more than 2.5 °F over baseline, or at least 1.5 °F over baseline and ≥ 103.5 °F. Hypothermia is defined as a temperature ≤3.5°F below baseline. Lymphocytopenia, thrombocytopenia, monocytopenia, neutropenia, eosinopenia, and basopenia are defined by a ≥35% drop in numbers of lymphocytes, platelets, monocytes, neutrophils, eosinophils, or basophils, respectively. Lymphocytosis, thrombocytosis, monocytosis, neutrophilia, eosinophilia, and basophilia are defined by a 100% or greater increase in numbers of lymphocytes, platelets, monocytes, neutrophils, eosinophils, or basophils, respectively. Hyperglycemia is defined as a 100% or greater increase in levels of glucose. Hypoglycemia is defined by a a ≥25% decrease in levels of glucose. Anemia is defined as a concurrent ≥25% decrease in erythrocyte count, Hct, and Hgb. Hypoalbuminemia is defined by a ≥25% decrease in levels of albumin. Hypoproteinemia is defined by a ≥25% decrease in levels of total protein. Hypoamylasemia is defined by a ≥25% decrease in levels of serum amylase. Hypocalcemia is defined by a ≥25% decrease in levels of serum calcium. Increases in ALT, AST, ALP, CRE, CRP, Hct, and Hgb were graded on the following scale: ↑ = 1-5 fold, ↑↑ = >5-10 fold, ↑↑↑ = >10-20 fold, ↑↑↑↑ = >20-fold, ↓ = ≥50% decrease. (BUN) blood urea nitrogen, (ALT) alanine aminotransferase, (AST) aspartate aminotransferase, (ALP) alkaline phosphatase, (CRE) Creatinine, (CRP) C-reactive protein, (Hct) hematocrit, (Hgb) hemoglobin.

Supplementary Table 2. Clinical description and outcome of rhesus macaques following SUDV challenge with remdesivir, MBP431, or combination treatment initiated 6 dpi.

NHP	Sex	Treatment	Clinical illness	Clinical pathology
D6-RDV-1	F	Remdesivir	Decreased appetite (d5); anorexia (d6-8); fever (d6); depression (d7, 8); petechial rash (d6-8); hunched posture (d7, 8); recumbency (d7, 8); unresponsiveness (d8); venipuncture hemorrhaging (d8). Subject euthanized (d8).	Lymphocytopenia (d6, 8); thrombocytopenia (d6, 8); monocytopenia (d8); eosinopenia (d8); basopenia (d8); eosinophilia (d6); hypoglycemia (d8); hypoalbuminemia (d6, 8); hypoamylasemia (d6); BUN ↑ (d8); CRE ↑ (d6, 8); ALT ↑ (d6, 8); AST ↑↑↑ (d6, 8); ALP ↑ (d6); GGT ↑ (d8); CRP ↑↑↑↑ (d6), ↑↑↑ (d8).
D6-RDV-2	F	Remdesivir	Decreased appetite (d6-9, 11-13, 15, 17); petechial rash (d6-12); anorexia (d10); depression (d10-12); hunched posture (d10-12); weakness (d10, 11); facial edema (d13-17); jaundice (15-20); keratic precipitate (d17, 19-33); conjunctivitis (d19, 20); ocular cloudiness (d19-22). Subject survived to study endpoint (d35).	Lymphocytopenia (d6); thrombocytopenia (d6, 9, 12); monocytopenia (d6, 9, 21, 35); lymphocytosis (d15); thrombocytosis (d28); monocytosis (d12, 15, 28); neutrophilia (d6, 9, 12, 15, 21); eosinophilia (d6, 9, 12, 15, 21, 28, 35); basophilia (d15, 21, 28, 35); hypoglycemia (d9, 12, 21); hypoalbuminemia (d9, 12, 15, 21, 28); hypoproteinemia (d9, 12, 15); hypoamylasemia (d6, 9); CRE ↑ (d6, 9); ALT ↑ (d6, 9, 12, 15, 21); AST ↑↑↑↑ (d6), ↑↑↑ (d9, 12, 15); ↑ (d21); ALP ↑ (d6, 9, 12, 15, 21, 28); GGT (d21, 28); CRP ↑↑↑↑ (d6), ↑↑ (d9).
D6-RDV-3	M	Remdesivir	Decreased appetite (5-8); fever (d6, 7); petechial rash (d7-9); anorexia (d9-11); depression (d10, 11); hunched posture (d10, 11); facial edema (d11); weakness (d11); recumbency (d11); unresponsiveness (d11). Subject euthanized (d11).	Lymphocytopenia (d6, 9, 11); thrombocytopenia (d6, 9, 11); monocytopenia (d9); eosinopenia (d9); basopenia (d9, 11); monocytosis (d6); neutrophilia (d11); hypoglycemia (d11); hypocalcemia (d6); hypoalbuminemia (d9, 11); hypoproteinemia (d11); hypoamylasemia (d6, 9, 11); ALT ↑ (d6, 9); AST ↑↑↑ (d6), ↑↑ (d9), ↑ (d11); GGT ↑ (d6); CRP ↑ (d9, 11).
D6-RDV-4	F	Remdesivir	Facial flushing (d1, 5); decreased appetite (d5, 6); hunched posture (d5, 6); weakness (d5, 7); depression (d6, 7); anorexia (d7); petechial rash (d6, 7); ecchymosis (d7); recumbency (d7); unresponsiveness (d7). Subject euthanized (d7).	Lymphocytopenia (d6); thrombocytopenia (d6, 7); monocytopenia (d6, 7); basopenia (d7); neutrophilia (d6, 7); eosinophilia (d6); hypocalcemia (d7); hypoalbuminemia (d6, 7); hypoamylasemia (d6); BUN ↑ (d6), ↑↑ (d7); CRE ↑ (d6), ↑↑↑ (d7); ALT ↑ (d6), ↑↑ (d7); AST ↑↑↑↑ (d6, 7); ALP ↑ (d6, 7); GGT ↑ (d7); CRP ↑↑↑↑ (d6, 7).
D6-RDV-5	M	Remdesivir	Decreased appetite (d1, 6); anorexia (d7-9); inguinal lymphadenitis (d6); petechial rash (d7-9); depression (d9); weakness (d9); hunched posture (d9); recumbency (d9); ataxia (d9); penile rash (d9); tachypnea (d9). Subject euthanized (d9).	Lymphocytopenia (d9); thrombocytopenia (d6, 9); basopenia (d9); monocytosis (d6); neutrophilia (d6); eosinophilia (d6), basophilia (d6); hypoalbuminemia (d6, 9); hypoamylasemia (d6, 9); BUN ↑ (d9); ALT ↑↑ (d9); AST ↑ (d6), ↑↑↑↑ (d9); GGT ↑ (d9); CRP ↑↑↑↑ (d6), ↑↑↑ (d9).
D6-MBP-1	M	MBP431	Fever (d6); decreased appetite (d6-10); petechial rash (d8-10); periorbital edema (d20-27); ocular cloudiness (d20-35); keratic precipitates (d21-25). Subject survived to study endpoint (d35).	Lymphocytopenia (d6, 9); thrombocytopenia (d6, 9, 12); monocytopenia (d9, 15, 21, 35); neutropenia (d28); eosinopenia (d9, 15); basopenia (d9); lymphocytosis (d12); monocytosis (d6); neutrophilia (d9, 12, 15, 21); eosinophilia (d6); basophilia (d21); hypoglycemia (d15); hypoalbuminemia (d9, 12, 15); hypoamylasemia (d6, 9, 12, 15, 21, 28, 35); ALT ↑ (d9); AST ↑ (d6, 9); ALP ↑ (d9); CRP ↑↑↑↑ (d6), ↑ (d9, 21).
D6- MBP -2	F	MBP431	Fever (d6); anorexia (d6); decreased appetite (d7-11); petechial rash (d6-11); hunched posture (d8-10); epistaxis (d8, 10, 11); depression (d8-11); periorbital edema (d10); weakness (d10, 11); recumbency (d11). Subject euthanized (d11).	Lymphocytopenia (d6, 9, 11); thrombocytopenia (d6, 9, 11); eosinopenia (d6); basopenia (d6, 9); monocytosis (d9, 11); neutrophilia (d9, 11); eosinophilia (d11); hypoglycemia (d9); hypoalbuminemia (d6, 9, 11); hypoproteinemia (d9); hypoamylasemia (d6, 9, 11); ALT ↑↑ (d9), ↑ (d11); AST ↑ (d6), ↑↑↑↑ (d9), ↑↑↑ (d11); ALP ↑ (d9, 11); GGT ↑ (d9); CRP ↑↑↑↑ (d6), ↑↑↑ (d9, 11).
D6- MBP -3	M	MBP431	Fever (d6); anorexia (d6-11); petechial rash (d8-11); depression (d10, 11); weakness (d10, 11); hunched posture (d10, 11); recumbency (d10, 11); ataxia (d11); unresponsiveness (d11). Subject euthanized (d11).	Lymphocytopenia (d6, 9, 11); thrombocytopenia (d6, 9, 11); basopenia (d9); monocytosis (d6, 11); neutrophilia (d6, 9, 11); eosinophilia (d6, 9, 11); basophilia (d11); hypoalbuminemia (d9, 11); hypoamylasemia (d6, 9); AST ↑ (d6); CRP ↑↑↑↑ (d6), ↑↑ (d9), ↑↑↑↑ (d11).

D6- MBP -4	F	MBP431	Fever (d6); decreased appetite (d6); anorexia (d7-9); petechial rash (d8, 9); weakness (d9); recumbency (d9); unresponsiveness (d9). Subject euthanized (d9).	Lymphocytopenia (d6, 9); monocytopenia (d6, 9); basopenia (d9); thrombocytosis (d6); neutrophilia (d6, 9); eosinophilia (d6, 9); hypoglycemia (d6, 9); hypoalbuminemia (d9); hypoproteinemia (d9); hypoamylasemia (d6, 9); BUN ↑ (d9); CRE ↑ (d9); ALT ↑↑ (d9); AST ↑ (d6), ↑↑↑ (d9); ALP ↑ (d9); GGT ↑ (d9); CRP ↑↑↑↑ (d6, 9).
D6- MBP -5	F	MBP431	Decreased appetite (d0-5); anorexia (d6-11); fever (d6); petechial rash (d8-11); inguinal lymphadenitis (d9); recumbency (d9-11); weakness (d10, 11); facial edema (d11). Subject euthanized (d11).	Lymphocytopenia (d6, 9, 11); thrombocytopenia (d6, 9, 11); monocytopenia (d6, 9, 11); basopenia (d9); neutrophilia (d6, 9, 11); eosinophilia (d6, 11); ↓ Hgb (d6, 9, 11); hypoalbuminemia (d9, 11); hypoamylasemia (d6, 9, 11); ALT ↑↑ (d9), ↑ (d11); AST ↑ (d6, 9, 11); ALP ↑ (d9, 11); GGT ↑ (d9); CRP ↑↑↑↑ (d6, 11), ↑↑↑ (d9).
D6-COMB-1	F	Remdesivir + MBP431	Decreased appetite (d1, 6-15); fever (d6); diarrhea (d6-14); loose stool (d15). Subject survived to study endpoint (d35).	Lymphocytopenia (d6, 9); thrombocytopenia (d6, 9, 12); monocytopenia (d9, 12, 15, 21, 28, 35); eosinopenia (d9, 12); basopenia (d9, 12, 28); neutrophilia (d6, 9, 12, 15, 28, 35); eosinophilia (d6); hypoalbuminemia (d9, 12, 15); hypoamylasemia (d6, 9); CRE ↑ (d6); AST ↑ (d12); CRP ↑↑↑↑ (d6), ↑ (d9).
D6-COMB-2	M	Remdesivir + MBP431	Fever (d6); decreased appetite (d6-9, 11-13); anorexia (d10). Subject survived to study endpoint (d35).	Lymphocytopenia (d6, 9); monocytopenia (d28); neutropenia (d12, 21, 28); monocytosis (d6, 12); neutrophilia (d6); eosinophilia (d6, 15); basophilia (d6); hypoamylasemia (d6, 9); CRP ↑↑↑↑ (d6), ↑↑↑ (d9).
D6-COMB-3	F	Remdesivir + MBP431	Anorexia (d6, 7, 10); decreased appetite (d8, 9, 11-13, 29); petechial rash (d7-12, 21, 22); periorbital edema (d28-35); conjunctivitis (d29-35); ocular cloudiness (d29-35); keratic precipitate (d34, 35). Subject survived to study endpoint (d35).	Lymphocytopenia (d6, 28); thrombocytopenia (d6, 9); monocytopenia (d12, 15, 21, 28, 35); neutropenia (d6, 9); eosinopenia (d9); basopenia (d9, 12); lymphocytosis (d21); neutrophilia (d28, 35); eosinophilia (d21, 28); basophilia (d28); hypoalbuminemia (d6, 9, 12, 15, 35); hypoamylasemia (d6, 9, 12); AST ↑ (d6, 9, 15); CRP ↑↑↑↑ (d6), ↑↑ (d28).
D6-COMB-4	M	Remdesivir + MBP431	Anorexia (d6, 7); facial flushing (d6, 7) decreased appetite (d8-12); petechial rash (d7, 8); recumbency (d8); weakness (d10); keratic precipitate (d19-30); conjunctivitis (d21); periorbital edema (d21, 22); ocular discharge (d21, 22). Subject survived to study endpoint (d35).	Lymphocytopenia (d6, 9); thrombocytopenia (d6, 9); neutropenia (d15, 28, 35); eosinopenia (d35); basopenia (d35); lymphocytosis (d12); monocytosis (d6, 12, 15, 21, 35); neutrophilia (d6, 9, 21); eosinophilia (d12, 21); basophilia (d21); hypoalbuminemia (d9, 12, 15, 21); hypoamylasemia (d6, 9); ALT ↑ (d9); AST ↑ (d6, 9); CRP ↑↑↑↑ (d6), ↑ (d9), ↑↑ (d21).
D6-COMB-5	F	Remdesivir + MBP431	Fever (d6-10); anorexia (d6, 9-11); depression (d10, 11); hunched posture (d10, 11); weakness (d10, 11); petechial rash (d11). Subject succumbed to disease (d12).	Lymphocytopenia (d6, 9); thrombocytopenia (d6, 9); monocytopenia (d9); basopenia (d9); monocytosis (d6); neutrophilia (d6, 9); eosinophilia (d6); hypoglycemia (d6); hypoalbuminemia (d9); hypoamylasemia (d6, 9); ALT ↑ (d9); AST ↑ (d6, 9); ALP ↑ (d9); GGT ↑ (d9); CRP ↑↑↑↑ (d6), ↑ (d9).
D6-CTRL	F	In-Study Control	Decreased appetite (d5); hunched posture (d5, 6); weakness (d5, 6); anorexia (d6); depression (d6); petechial rash (d6); ecchymosis (d6); unresponsiveness (d6). Subject euthanized (d6).	Lymphocytopenia (d6); thrombocytopenia (d6); monocytosis (d6); neutrophilia (d6); eosinophilia (d6); hypocalcemia (d6); hypoalbuminemia (d6); hypoamylasemia (d6); BUN ↑ (d6); CRE ↑↑ (d6); ALT ↑↑ (d6); AST ↑↑↑↑ (d6); ALP ↑ (d6); GGT ↑↑ (d6); CRP ↑↑↑↑ (d6).

Days after SUDV challenge are in parentheses. All reported findings are in comparison to baseline (d0) values. Decreased appetite is defined as some food but not all food consumed from the previous day. Anorexia is defined as no food consumed from the previous day. Fever is defined as a temperature more than 2.5 °F over baseline, or at least 1.5 °F over baseline and ≥ 103.5 °F. Hypothermia is defined as a temperature ≤3.5°F below baseline. Lymphocytopenia, thrombocytopenia, monocytopenia, neutropenia, eosinopenia, and basopenia are defined by a ≥35% drop in numbers of lymphocytes, platelets, monocytes, neutrophils, eosinophils, or basophils, respectively. Lymphocytosis, thrombocytosis, monocytosis, neutrophilia, eosinophilia, and basophilia are defined by a 100% or greater increase in numbers of lymphocytes, platelets, monocytes, neutrophils, eosinophils, or basophils, respectively. Hyperglycemia is defined as a 100% or greater increase in levels of glucose. Hypoglycemia is defined by a a ≥25% decrease in levels of glucose. Anemia is defined as a concurrent ≥25% decrease in erythrocyte count, Hct, and Hgb. Hypoalbuminemia is defined by a ≥25% decrease in levels of albumin. Hypoproteinemia is defined by a ≥25% decrease in levels of total protein. Hypoamylasemia is defined by a ≥25% decrease in levels of serum amylase. Hypocalcemia is defined by a ≥25% decrease in levels of serum calcium. Increases in ALT, AST, ALP, CRE, CRP, Hct, and Hgb were graded on the following scale: ↑ = 1-5 fold, ↑↑ = >5-10 fold, ↑↑↑ = >10-20 fold, ↑↑↑↑ = >20-fold, ↓ = ≥50% decrease. (BUN) blood urea nitrogen, (ALT) alanine aminotransferase, (AST) aspartate aminotransferase, (ALP) alkaline phosphatase, (CRE) Creatinine, (CRP) C-reactive protein, (Hct) hematocrit, (Hgb) hemoglobin.

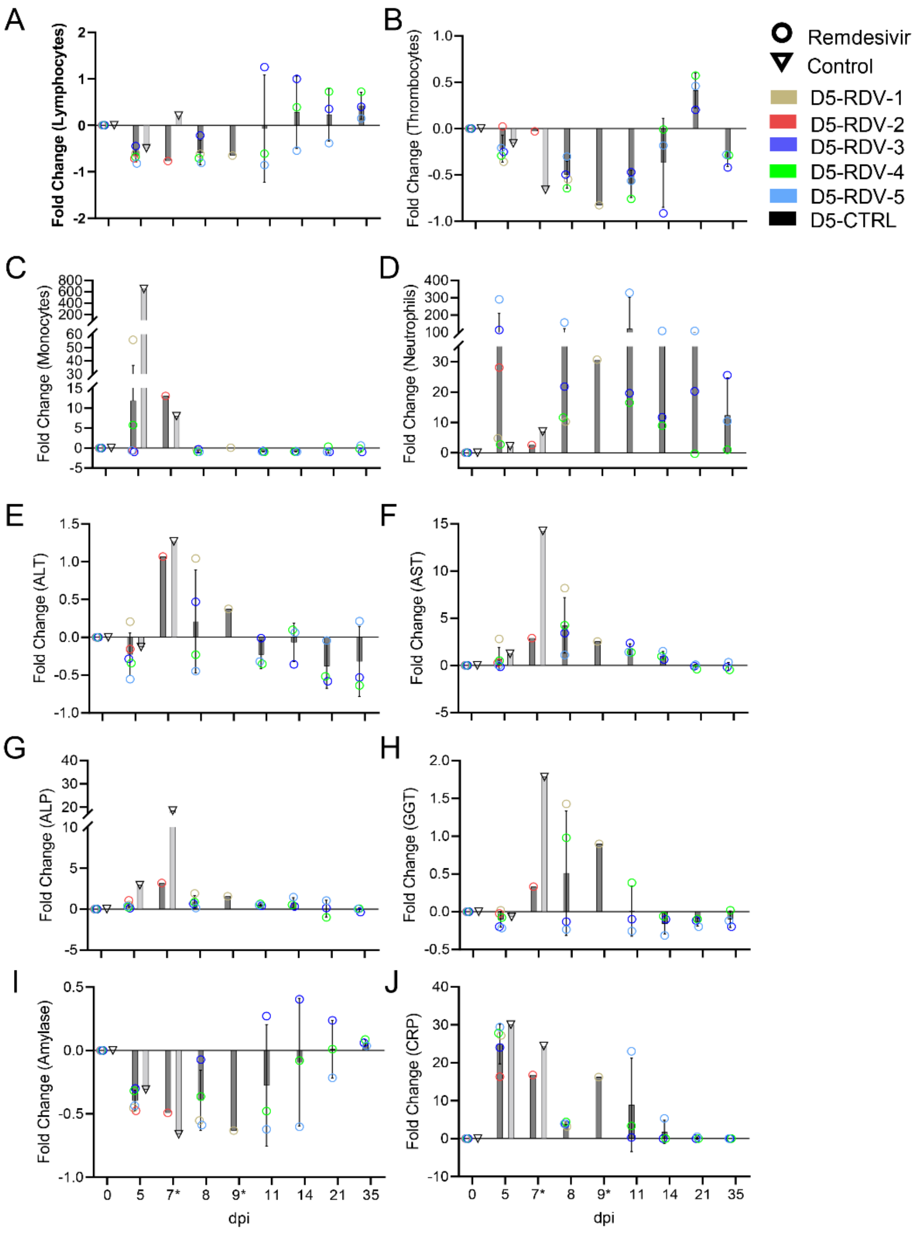

Supplementary Figure 1: Hematological profiles of SUDV-challenged rhesus macaques receiving remdesivir treatment beginning 5 dpi. Whole blood counts and serum biochemistry analyses were performed on blood/serum obtained from each subject on the indicated days post-challenge. For all panels, data is presented as fold-change from individual subject baseline (0 dpi) values. Bars indicate the mean for each cohort at the indicated timepoint, error bars represent ±

SD. Values for individual animals within each cohort are shown as color-coded symbols. Days marked with asterisks on the x-axis denote terminal timepoints for the indicated subject(s).

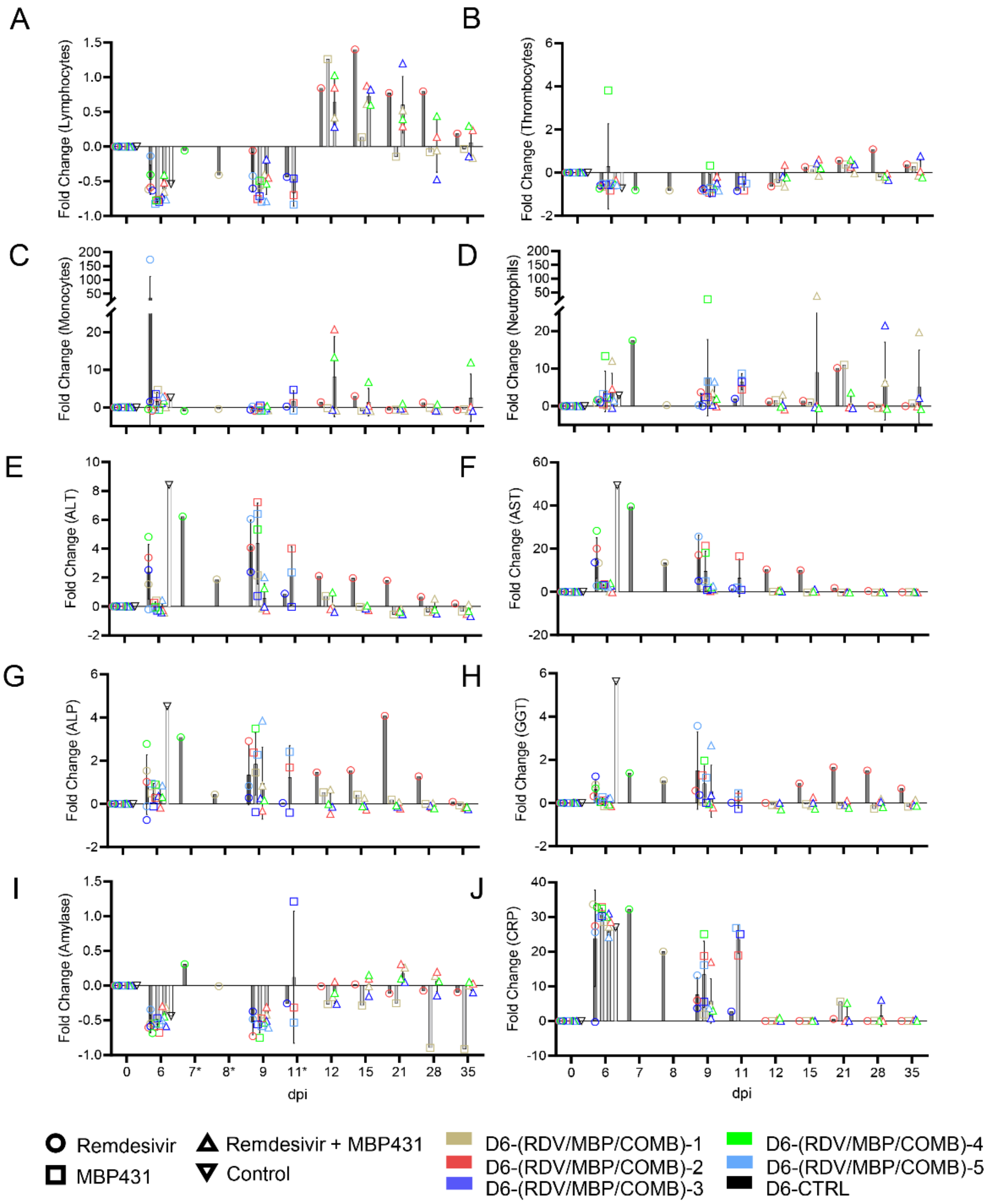

Supplementary Figure 2: Hematological profiles of SUDV-challenged rhesus macaques receiving remdesivir treatment beginning 6 dpi. Whole blood counts and serum biochemistry analyses were performed on blood/serum obtained from each subject on the indicated days post-

challenge. For all panels, data is presented as fold-change from individual subject baseline (0 dpi) values. Bars indicate the mean for each cohort at the indicated timepoint, error bars represent ± SD. Values for individual animals within each cohort are shown as color-coded symbols. Days marked with asterisks on the x-axis denote terminal timepoints for the indicated subject(s).

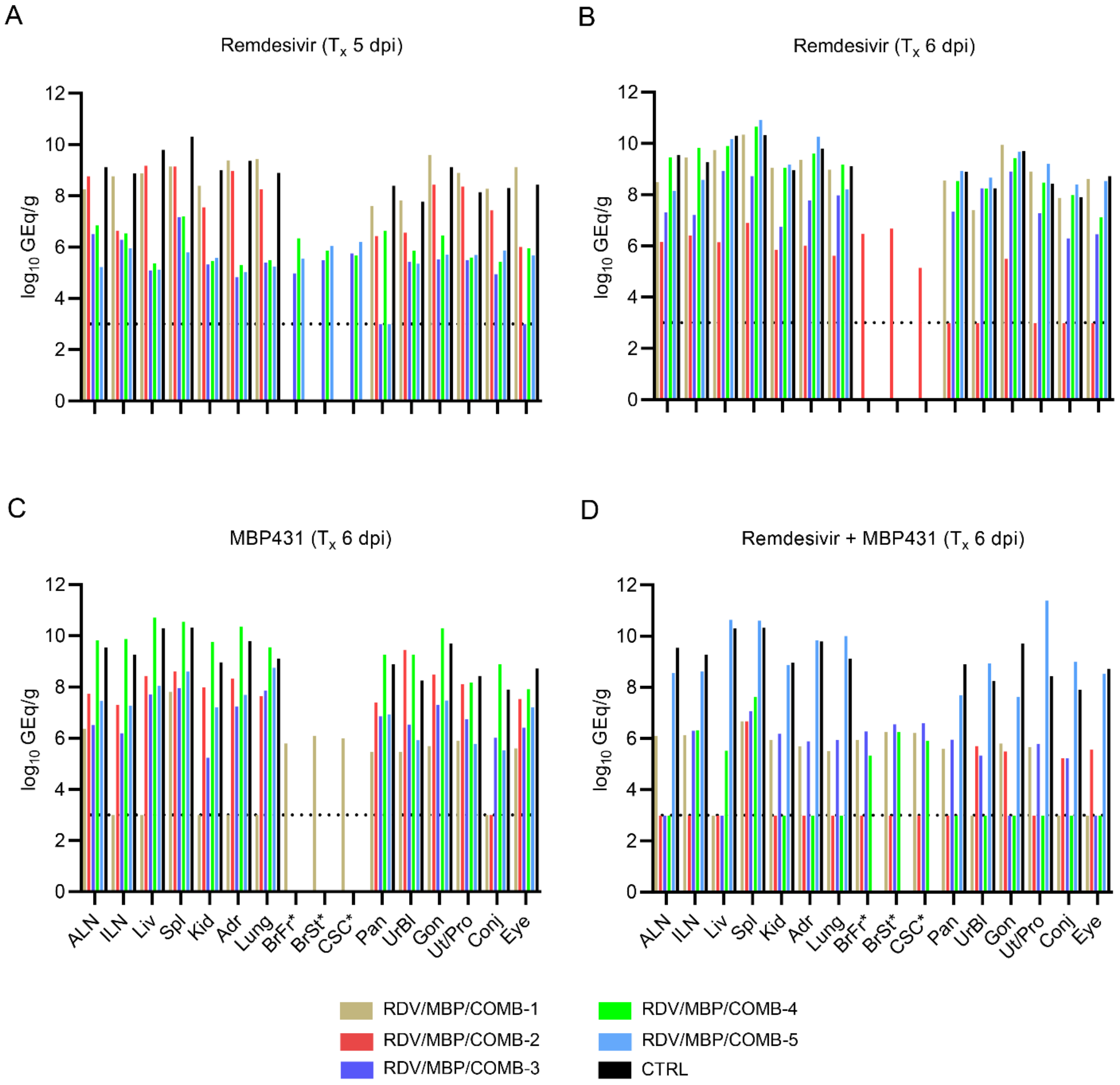

Supplementary Figure 3: Viral load in tissues from SUDV-challenged rhesus macaques. Viral load was determined by RT-qPCR of RNA from selected tissues harvested at necropsy. **(A)** treatment 5 dpi with remdesivir, (**B**) treatment 6 dpi with remdesivir, **(C)** treatment 6 dpi with MBP431, **(D)** or combined treatment 6 dpi with remdesivir/MBP431. For all panels, individual bars represent the mean of two technical replicates. Dashed horizontal lines indicate the limit of detection (LOD) for the assay (1000 GEq/mL). "*" next to the tissue name indicates that the tissue was not collected or assayed for all subjects. Subjects for which values were below LOD are plotted as 999 GEq/mL to differentiate from subjects for which a given tissue was not collected or analyzed. for that subject. Abbreviations for tissues: ALN: Axillary lymph node; ILN: inguinal lymph node; Liv: liver; Spl: spleen; Kid: kidney; Adr: adrenal gland; BrFr: brain frontal cortex; BrSt: brain stem; CSC: cervical spinal cord; Pan: pancreas; UrBl: urinary bladder; Gon: gonad; Ut/Pro: uterus/prostate; Conj: conjunctiva.
